# Auditory Countermeasures for Sleep Inertia: Exploring the Effect of Melody and Rhythm in an Ecological Context

**DOI:** 10.3390/clockssleep2020017

**Published:** 2020-05-29

**Authors:** Stuart J. McFarlane, Jair E. Garcia, Darrin S. Verhagen, Adrian G. Dyer

**Affiliations:** 1 School of Media and Communication, RMIT University, Melbourne 3001, Australia; jair.garcia@rmit.edu.au (J.E.G.); adrian.dyer@rmit.edu.au (A.G.D.); 2 School of Design, RMIT University, Melbourne 3001, Australia; darrin.verhagen@rmit.edu.au

**Keywords:** sleep inertia, auditory perception, sustained attention, human performance, alarm tones, music, melody, rhythm

## Abstract

Sleep inertia is a decline in cognition one may experience upon and following awakening. A recent study revealed that an alarm sound perceived as melodic by participants displayed a significant relationship to reports of reductions in perceived sleep inertia. This current research builds on these findings by testing the effect melody and rhythm exhibit on sleep inertia for subjects awakening in their habitual environments. Two test Groups (A and B; *N* = 10 each) completed an online psychomotor experiment and questionnaire in two separate test sessions immediately following awakening from nocturnal sleep. Both groups responded to a control stimulus in the first session, while in the second session, Group A experienced a melodic treatment, and Group B a rhythmic treatment. The results show that the melodic treatment significantly decreased attentional lapses, false starts, and had a significantly improved psychomotor vigilance test (PVT) performance score than the control. There was no significant result for reaction time or response speed. Additionally, no significant difference was observed for all PVT metrics between the control–rhythmic conditions. The results from this analysis support melodies’ potential to counteract symptoms of sleep inertia by the observed increase in participant vigilance following waking from nocturnal sleep.

## 1. Introduction

Sleep inertia (SI) is a transitional sleep–wake phenomenon defined by a reduction in human performance upon and post awakening [1,2,3]. The adverse features of SI have been shown to protract for approximately 0–30 min post awakening, however, durations spanning up to 4 h have also been reported [3,4,5,6,7,8,9]. Studies have shown SI to impair several dimensions of cognitive performance, including reaction time (RT) [10,11,12], and decision making [13,14]. In a real-world context, Wertz et al. [4] suggest that the resulting decline in performance may be on par, or more pronounced, than being legally intoxicated and/or having a night of complete sleep deprivation.

Countermeasures for SI [1,3,15] have been researched through several contexts and themes, and may be considered as extensions or augmentations of ubiquitous human behaviours, environmental conditions, and routines. These include caffeine [10,16,17,18,19], light [12,18,20,21,22], temperature [23,24], post-awakening routines [18,25], sound [26,27,28], and stress [29].

Concerning this current research investigation, three previous studies have been identified that examine how audio may impact SI post awakening. Tassi et al. [26] concluded that pink noise (75 dB) can reduce SI when deployed as an intense waking alarm, while Hayashi [27] detected that excitatory music, particularly high-preference popular music (60 dB) as specified by participants has the potential to reduce the impact of SI after a short nap. Through an ecologically valid approach, McFarlane et al. [28] revealed that participant alarm sounds perceived as melodic showed a significant relationship to reductions in perceived sleep inertia, as compared to their “neither unmelodic nor melodic” counterparts. Additionally, it was shown that a melodic alarm sound is perceived to be more rhythmic than a neutral interpretation [28]. Taken together, the studies discussed above demonstrate that sound and music are plausible awakening countermeasures for SI, and, with additional research, we may establish a refined understanding of the auditory aesthetics and musical mechanisms required for the best-practice design of such stimuli.

From an operational and end-user perspective, real-world circumstances may benefit from sound stimuli targeted at the reduction of SI, including occupational settings; where audio is employed to activate employees immediately following awakening, and improved day-to-day awakening alarm tones, which are common within society and our auditory ecology [30].

In the current study, we examine the influence saliently melodic and rhythmic alarm tone treatments have on SI immediately post awakening in ecological conditions comparative to a non-melodic control. Through this investigation, we aim to build on initial findings by McFarlane et al. [28] to further understand whether and how melody may assist in counteracting SI, and the relationship rhythm may have to SI reduction when tested in isolation. Within the broader context of auditory-assisted awakening, our goal is to provide evidence that may be expanded upon and referenced in the future development and testing of sound stimuli to counteract SI in both natural, auditory complex surroundings and laboratory conditions.

## 2. Results

### 2.1. Test Group A: Melody Stimulus

Participants were aged between 18 and 49 (18–29 (*n* = 5), 30–39 (*n* = 2), 40–49 (*n* = 3)), consisted of males and females (40% female), with 40% of participants reporting consistent sleep epochs of 5–7 h per night, and 50% reporting 7+ hours respectively (3–5 h (*n* = 1), 5–7 h (*n* = 4), 7–9 h (*n* = 4), 9+ hours (*n* = 1)).

**Figure 1:**
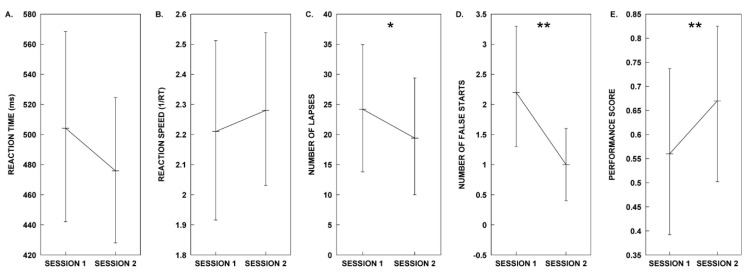
Test Group A plots for psychomotor vigilance test (PVT) metrics in each test session. Results for the five different PVT metrics obtained from participants in Group A during sessions 1 and 2. (**A**) mean reaction time, (**B**) mean reaction speed, (**C**) mean number of lapses, (**D**) mean number of false starts, and (**E**) mean performance score. We identified significant differences in performance at *α* = 0.05 for lapses, false starts, and performance. The *p*-values less than 0.05 are represented by (*). The *p*-values less than 0.01 are represented by (**). All error bars represent 95% confidence intervals. Refer to the Results section for details on the outcome of the statistical analyses.

The planned sequence of five paired-sample *t*-tests was performed to analyse the PVT metrics (mean RT, mean 1/RT, lapses, false starts, and performance score) within subjects for the control (Session 1) and melody (Session 2) conditions (See Figure 1. for plots of Group A’s PVT metrics in each test session). Our results show that there is no significant difference with a medium effect (*d* ≥ 0.5) [31] in the mean RT for the control and melody conditions, nor a significant difference between conditions with a small effect (*d* ≥ 0.2) [31] in mean inverse reaction time (mean 1/RT). The analysis does reveal a significant difference with a large effect (*d* ≥ 0.8) [31] between the mean lapses, mean false starts, and the PVT performance score results for the control and melody treatments. Specifically, the melody treatment resulted in superior performance considering lapses, false starts, and the PVT performance score (see Table 1).

**Figure 2:**
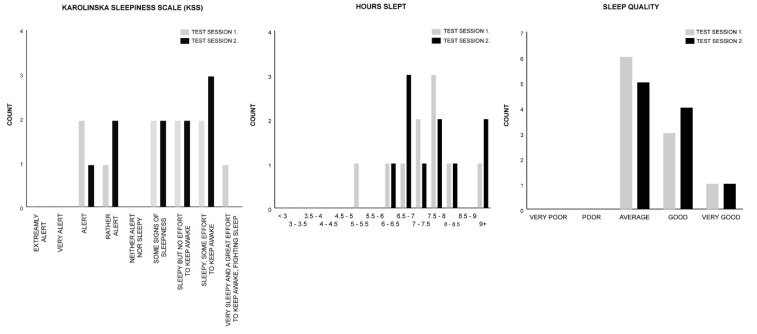
Test Group A histogram counts for the Karolinska sleepiness scale (KSS), hours slept, and sleep-quality measures.

The Wilcoxon signed rank test was utilized to individually analyse the median difference of the Karolinska sleepiness scale (KSS), hours slept, and sleep-quality measures within subjects for the control (Session 1) and rhythm (Session 2) conditions of Test Group A (See Figure 2. for the histogram counts of the KSS, hours slept, and sleep-quality measures). For all measures, the median Session 2 test ranks are not significantly different than the Session 1 test ranks, indicating that in both test sessions participants’ subjective sleep attributes are analogous (see Table 2).

Mean percentages across the two test sessions for each subjective measure show that 70% of participants reported ratings of “sleepiness” (some signs of sleepiness (20%); sleepy but no effort to keep awake (20%); sleepy, some effort to keep awake (25%); very sleepy and a great effort to keep awake, fighting sleep (5%)), while 30% reported being “rather alert” (15%) to alert (15%). The mean hours slept before each test session reveal that 65% of participants slept 7+ hours (6+ hours (95%)); and 100% reported ratings from “average” to “very good” sleep quality (average (55%); good (35%); very good (10%)).

### 2.2. Test Group B: Rhythm Stimulus

Participants were aged between 18 and 49 (18–29 (*n* = 4), 30–39 (*n* = 5), 40–49 (*n* = 1)), consisted of males and females (50% female), and reported a typical night’s sleep ranging from 5 to 9 h (5–7 h (*n* = 5; 50%), 7–9 h (*n* = 5, 50%)).

**Figure 3:**
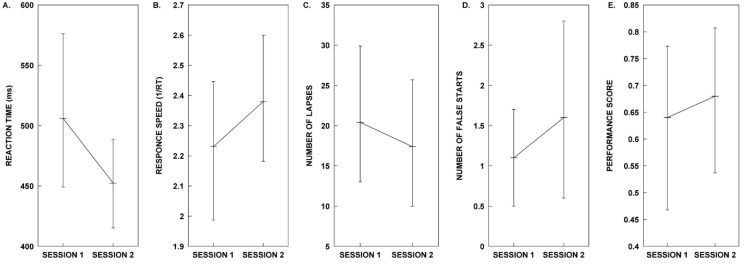
Results for the five different PVT metrics obtained from participants in Group B during Sessions 1 and 2. (**A**) Mean reaction time, (**B**) mean reaction speed, (**C**) mean number of lapses, (**D**) mean number of false starts, and (**E**) mean performance score. All error bars represent 95% confidence intervals. Refer to the Results section for details on the outcome of the statistical analyses.

Consistent with Test Group A’s analysis, planned paired-sample *t*-tests were performed to analyse the PVT metrics within subjects for the control (Session 1) and rhythm (Session 2) conditions (See Figure 3. for plots of Group B’s PVT metrics in each test session). Our results show that there is no significant difference in the mean RT, mean 1/RT, lapses, or performance score for the control and rhythm conditions. The Wilcoxon signed ranks test for false starts indicated that the median Session 2 test ranks were not significantly different from the Session 1 test ranks (Table 3).

**Figure 4:**
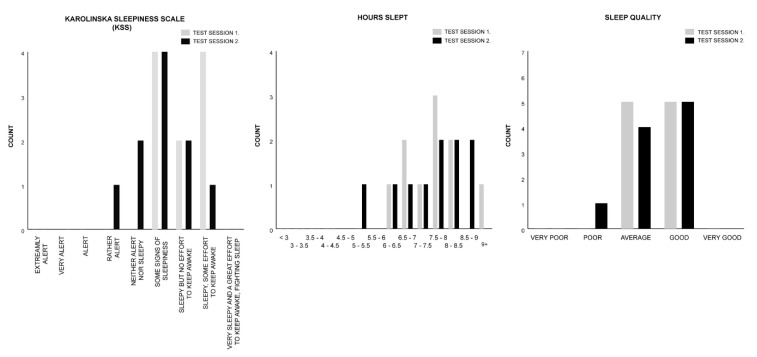
Test Group B histogram counts for the KSS, hours slept, and sleep-quality measures.

The Wilcoxon signed rank test was utilized to individually analyse the median difference of the KSS, hours slept, and sleep-quality measures within subjects for the control (Session 1) and rhythm (Session 2) conditions of Test Group B (See Figure 4. for the histogram counts of the KSS, hours slept, and sleep-quality measures). For all measures the median Session 2 test ranks were not significantly different from the Session 1 test ranks, indicating that in both test sessions participants’ subjective sleep attributes are comparable (see Table 4).

Group B’s mean percentages across both test sessions for each sleep measure show that 85% of all participants reported ratings of “sleepiness” (some signs of sleepiness (40%); sleepy but no effort to keep awake (20%); sleepy, some effort to keep awake (25%)). One hundred per cent (100%) of Session 2 participants reported a minimum rank of “sleepiness”. Thirty percent of Session 1 participants reported not to be sleepy (“neither alert nor sleepy” (20%); rather alert (10%)). Seventy percent (70%) of all Group B participants slept 7+ hours (6+ hours (95%)); and 95% reported ratings from “average” to “good” sleep quality (average (45%); good (50%)).

## 3. Discussion

The objective of this study was to assess the effect musically melodic and rhythmic auditory alarm tone treatments exhibit on symptoms of SI following awakening within ecological conditions. To date, this research represents the first experiment to test and report reproducible alarm tones with strategically composed melodic and rhythmic contours in the context of SI. The results obtained present preliminary insights that may be utilized to extend our understanding for how alarm tone design may assist to counteract SI, which may prove beneficial in scenarios where sustained attention is vital immediately upon waking. Examples could include land-based, aeronautical, and nautical transportation; critical monitoring tasks; or common day to day activities such as driving or riding a bike post awakening.

The principal results of this study reveal two key findings: (i) A saliently melodic alarm tone, incorporating a musically neutral control test stimulus within its design, enhanced vigilance post awakening when compared to the control stimulus in isolation. (ii) A rhythmic alarm tone comprising of the identical rhythmic contour as the melodic stimuli with the control embedded did not produce any significant difference between PVT performance metrics when verified against the control.

There was no significant difference in mean RT or mean 1/RT between the melodic stimuli and the control (mean RT: *p* = 0.184; mean 1/RT: *p* = 0.513), however, the melodic stimuli did produce a significant reduction in lapses, false starts, and returned a significantly superior PVT performance score than the control (lapses: *p* = 0.016; false starts: *p* = 0.001; PVT performance score: *p* = 0.006). These ecologically validated results demonstrate that the participants’ sustained attention had been significantly improved in the melodic condition upon awakening, suggesting that alarm tones (not to fast nor to slow; 105 Beats per minute (BPM) with melodically rhythmic features may be more successful in counteracting the deficits experienced from SI post awakening than counterparts devoid of melodic content (i.e., the control stimulus we tested).

The rhythmic alarm tone did not produce any significant difference between PVT performance metrics compared to the control (mean RT: *p* = 0.193; mean 1/RT: *p* = 0.318; lapses: *p* = 0.609; false starts: *p* = 0.194; PVT performance score: *p* = 0.683), revealing that a saliently rhythmic alarm sound devoid of melody may be equally effective as a monotonous tonal beat with respect to RT and sustained attention upon awakening.

We failed to reject the null hypothesis of equality between the subjective sleep attributes gathered for Test Group A test sessions (KSS: *p* = 1.000; hours slept: *p* = 0.236; sleep quality: *p* = 0.705); thus, rendering each test session comparable. We interpret this data to reveal that during each test session there is no global effect of chronic sleep deprivation with respect to participant performance. However, considering the variability of individual differences as measured by the subject’s typical hours slept per night, and hours slept prior to each test session, partial sleep deprivation may be present [32]. The mean percentages of all participants show that 50% report maintaining sleep epochs consistent with current recommendations (7+ hours per night), while 40% regularly sleep within or marginally below the “maybe appropriate” range (5–7 h) [33]. Prior to each test session, 65% of participants slept in the recommended range (7+ hours) and 95% of all subjects slept “maybe appropriate” (5–7 h) [33]. The subjective sleep quality ratings show all participants (100%) report to have had at least an “average” (~50%) to “very good” night’s sleep.

The KSS reports do provide evidence of mild SI upon awakening in both test sessions (70% did not require any effort to stay awake upon arousal). This would be inconsistent with our PVT data if the melody condition were assumed to be immediately effective upon awakening compared to the control. However, due to the mild state of SI observed, the KSS may not be as sensitive to the effects auditory stimuli may elicit on vigilance comparative to the PVT. For example, Kaida and Abe [34] reported that when testing alert participants during a monotonous task while exposed to an “own name” auditory condition, PVT Lapses were significantly improved relative to the other test conditions (including silent control), though there were no significant results observed in the KSS ratings to reflect the PVT data [34].

Similarly to Test Group A, Test Group B’s (rhythm, control) self-reported sleepiness and sleep data were not significantly different between test sessions (KSS: *p =* 0.089; hours slept: *p* = 0.666; sleep quality: *p* = 0.564), with the results indicating that it is unlikely participants in each test session exhibit chronic sleep deprivation. For example, 95% of participant sleep bouts preceding each test session may be suitable [33], with a majority (70%) reporting adequate sleep (7+ hours). The KSS and sleep-quality measures do show effects of SI as the majority of participants were sleepy upon arousal and ~50% reported an “average” night’s sleep.

The results obtained from this study present new evidence for the potential of auditory alarms to effectively counteract the inhibiting symptoms of SI in ecological conditions. Specifically, this study’s results support previous research showing a significant relationship between the reported melodicity of participants’ waking sound and a measure of perceived SI reduction [28].

One hypothesis we present for the lack of significant mean differences between the melody and control mean RT and mean 1/RT may be attributed to the beat intervals and perceived pace of both stimuli. In this study, the metre, beat, and tempo of each stimulus were identical. We hypothesize that by retaining the existing tempo and increasing the beat intervals of the control treatment within the melody stimulus may produce a rise in the perceived “pace” of the treatment, and in turn, affect response time in a similar manner that faster tempo music has been shown to reduce RT [35,36,37] when compared to slower music. We extend this hypothesis to the rhythm and control results in Test Group B also.

Reductions in mean lapses, false starts, and performance score between the control and melody treatments in this study are significant and may be attributed to the disparity in melodic content between the two stimuli. The control stimulus was designed specifically as an unmelodic counterpart to the melodic stimuli. This was achieved by retaining the control as the beat of the phrase, and strategically composing a melodic contour around the control. Previous research has shown that musical stimuli with melodic features (e.g., Vivaldi’s Four Seasons “Spring” concerto) increase task performance when compared to silent conditions [38,39], and it is posited by Riby [38] that this may be a consequence of music’s ability to increase arousal and enhance cognition. Additionally, the mode in which the music is sounded has been attributed to improvements in performance [40], and that pitches in the range of the female human voice (~<2500 Hz) may be more successful in arousing sleeping humans than those in the male range [41]. The frequency range of the melodic treatment in this study resides between 1318.5 and 2637 Hz.

The contrast between the control and melodic stimuli in this study are consistent with these findings and may, therefore, have contributed to the improved vigilance of participants post awakening. Similarly, this examination may clarify the lack of significance between the control and rhythm stimuli. The rhythmic treatment was composed to explore the effects of rhythm on SI in isolation, void of a melodic contour. In this regard, the absence of melody may account for the insignificant results against the control.

Field studies such as the one presented in this manuscript do provide valuable results with ecological validity, however, several limitations arise when compared to controlled laboratory investigations. For example, in uncontrolled test environments, researchers cannot guarantee conditions remain constant during each sleep bout. Thus, to accurately record environmental factors that may affect sleep quality (e.g., ambient temperature, light, and noise) several measurements would need to be recorded periodically during each participants sleep episode, which would occur when each participant is required to be sleeping. This presents technological challenges with participant, self-managed data procurement, logging, and reporting; particularly in the context of the research presented here. Managing and controlling for the effects of sleep deprivation on SI may also be considered a limitation to overcome. Although in this study all participants did report to maintain consistent sleep patterns, which is largely witnessed over the course of the experiment, more reliable data may be obtained by employing actigraphy and potentially prescribing stringent participant sleep–wake schedules (e.g., a 22.00–06.00; 8 h sleep episode). A protocol such as this would further control for sleep deprivation, however, by doing so, may sacrifice the ecological validity of the data obtained. Similarly, polysomnographic-verified awakenings are a robust method to analyse sleep-stage upon awakening, which would strengthen the interpretations and results provided in this study. However, the use of such devices may also confound the ecological validity of the results. For example, the process in which the hardware of these devices is applied to participants may be considered intrusive, and the wearing of the apparatus does not typically replicate real-world conditions in which humans sleep and wake. This may attribute to participant anxiety and/or disrupt sleep [42]. Again, actigraphy may be an appropriate trade-off against polysomnography in context, however, research suggests that actigraphy is limited in providing robust sleep-stage detection [43], and is also dependent on the type of device employed.

Future investigations into auditory countermeasures for SI may include the effects of tempo and melody, mode, or extend to additional auditory types including noise and the human voice. With respect to ecological inquiries the development of new methods and tools would assist in clarifying participant sleep profiles more efficiently and would be advantageous in studies where controlled laboratory conditions are unsuitable or unattainable (e.g., the International Space Station).

This research presents evidence demonstrating that a musically, melodically rhythmic alarm tone improves vigilance immediately upon awakening from a typical night’s sleep when compared to a metronomic alarm devoid of melody and with a restrained rhythmic contour. Additionally, this study’s results support previous research by McFarlane et al. [28] showing a significant relationship between the reported melodicity of a participants’ waking sound and a measure of perceived SI [28] in ecological conditions. Taken together, these results highlight the potential importance of musical melody in waking sound design as an agent to counteract SI, and more broadly emphasizes the requirement for research that tests musical elements of stimulus design beyond the broader, more general music classifications such as genre.

Auditory alarms are a popular tool for assisted awakening. Our research provides preliminary evidence that may be utilized to produce effective auditory designs to counteract the unfavourable effects of SI for improved day-to-day awakening. In this study, we thus provide the material required to accurately synthesize the stimuli we have employed, enabling future methodologies to further explore melody’s effect on SI.

## 4. Materials and Methods

### 4.1. Ethics Statement

All research methods, participant numbers (*N* = 20) considered appropriate for the study, and data collection were approved by the Royal Melbourne Institute of Technology University’s (RMIT) College of Human Ethics Advisory Network (CHEAN) (ref: CHEAN B 21753-10/18). The software application G*Power [44,45] was used to determine sample size by performing an a priori power analysis for large effects (Cohen’s *d* = 0.90) [46] for the paired *t*-test employed in this study. Our number of subjects was also consistent with Hayashi and Uchida [27] and associated research conducted by Horne and Moseley [14], and Smith and Kilby [47]. Respondents provided their specific consent to participate by completing the online study. This was stipulated to the subjects in the “invitation to participate” email distributed during the recruitment period and reiterated prior to undertaking the online test. The study was launched during May 2019 and concluded in November 2019.

### 4.2. Participants

Subjects were invited to participate through RMIT’s School of Media and Communications staff, student, and membership networks, printed posters located throughout the RMIT University Melbourne city campus, and through the researchers’ social networking communities. Individuals interested in volunteering for the study contacted the lead researcher directly via email. The volunteers were then supplied the study’s “invitation to participate” form. The contents included an introduction, title and overview of the research, who is conducting the study, participants’ rights and responsibilities, instructions for how to undertake the test, and contact information for any further enquiries regarding the test. Ideal participants were required to be 18 years and above, healthy with good hearing, have a consistent sleeping pattern and access to a smartphone, computer, tablet, or laptop, and a secure internet connection. Participants where not remunerated for their service. All eligible participants were encouraged to undertake the study without bias towards music preference or training.

The recruited participants’ gender classification was obtained through a male, female, and X (intermediate/intersex/unspecified) question in reference to the Australian Government guidelines on recognition of sex and gender [48]. Further, an option of non-disclosure was included to accommodate any participant willing to volunteer, yet reticent in recording any gender classification (i.e., prefer not to disclose). We have refrained from reporting or supplying the gender demographics for each individual analysis of the participants as open access data to support data minimization. This research reporting strategy is consistent with the General Data Protection Regulation (GDPR) [49].

### 4.3. Data Collection

The reported data were captured digitally via the use of the online software system Gorilla [50], where the questionnaire and experiment was contained, managed, and remotely accessed. Gorilla is software specifically produced for the undertaking of online questionnaires and experiments enabling researchers to design and implement their studies for ethically compliant distribution and data collection. The data obtained through Gorilla are securely stored and available for download and analysis by researchers. Gorilla is fully compliant with GDPR [50], and is developed with reference to The British Psychological Society [51], and National Institute for Health Research [52] standards.

### 4.4. Test Stimulus—Design and Description

All stimuli are in the key of C, have a metre of 4:4, a tempo of 105 BPM, and comprise a monophonic (control) and polyphonic (melody, rhythm) texture. The arrangements were designed as a two-bar motif and repetitively looped to a total duration of 108 s for Android or PC users, and 2 bars for Apple users due to the specific audio playback features of each platform. All compositions were produced in the audio production software package Cakewalk [53] and employed the TTS-1 soft synth to trigger the Vibraphone W and Woodblock timbral sample sets from the sound library provided. The final compositions were digitally limited and compressed to produce clear and balanced audio, which was exported as MP3/4 files. Caution was applied during the design phase to the auditory performance of the stimuli when relayed through various multimedia devices (e.g., mobile phones, laptops, and tablets). Lower frequencies do not perform as effectively as higher tones due to the limited frequency range these device types can produce [54]. All stimuli were iteratively prototyped through extensive field testing during the design development period (2018–2019).

The objective for the auditory design of the control, melodic, and rhythmic test stimulus was to produce a set of three recognizable, yet original complementary compositions, that when qualitatively compared, were differentiated and easily interpreted by their individual musical attributes (i.e., control (neither overtly rhythmic nor melodic); melodic, and rhythmic). In this way, we established a framework where the stimuli designed, in conjunction with the experimental study design, enabled the elemental analysis of each stimulus and its effect on SI.

To achieve this, we first produced the control as a metronomic pulse that could be sounded independently and perform as the “heartbeat” to both the melodic and rhythmic stimulus. The melodic and rhythmic stimuli were layered upon the control and strategically composed to accentuate their elemental musical aesthetics by means of timbre and contour. See Table 5 for the design overview of the stimulus set.

The key of C was deemed appropriate for the three stimuli in this study’s context considering its extensive application in popular music and its universal familiarity [55,56]. Similarly, the 4:4 metre, also known as “common time” [57], was selected as it is the most frequently employed and recognisable time signature in Western music today [56,58,59].

A tempo of 105 BPM was established as an appropriate pace for the function of the stimuli we sought to achieve, which is to successfully enable awakening, yet not to be overtly alarming, salient or fast, or slow or calming. Residing in the range of the classical andante tempi (76–108 BPM) [60], the preferred perceptual tempo (PPT) (also identified as preferred tempo and indifference interval) [61,62] of 100 BPM as proposed by Fraisse [62] and marginally slower than Moelants’ [61] finding of 120 BPM. A tempo of 105 BPM may be described as an approximate “mid-range” with respect to the human tempo registration range (existence region) of 40–300 BPM [63]. This method was chosen to allow for the targeted within-subject comparisons between the respective musical treatments without the influence of tempo (slow-paced vs. fast-paced) on arousal.

The control stimulus comprises two timbres (Woodblock, Vibraphone) that are sounded on every first and third beat of each bar. The Woodblock timbre is the percussive element of the score and is denoted the single note D6 with respect to an 88 key virtual piano (100% velocity: 240 ms duration). The tonal element of the control layers the Vibraphone sample as a single C6 note in relationship to an 88 key virtual piano (100% velocity: 240 ms duration) over the D6 percussive notes. When played, the control produces a metronomic and inexpressive pulse.

The melodic stimuli retains the D6 (100% velocity; 240 ms) percussive timbre and arrangement of the control, yet strategically increases the vertical tonal contour and horizontal rhythmic contour of the Vibraphone W notes within the composition. By introducing musical notes C7, A6, G6, E7, and E6 to the control, reducing inter-onset intervals (IOI’s) between notes, and enhancing the tonal contour, the resulting passage was designed to generate a dramatic rise in perceived melodicity when compared to the control. The dynamic aesthetic of this composition employs variations in note velocity (85–100%), and duration (174–340 ms).

**Figure 5:**
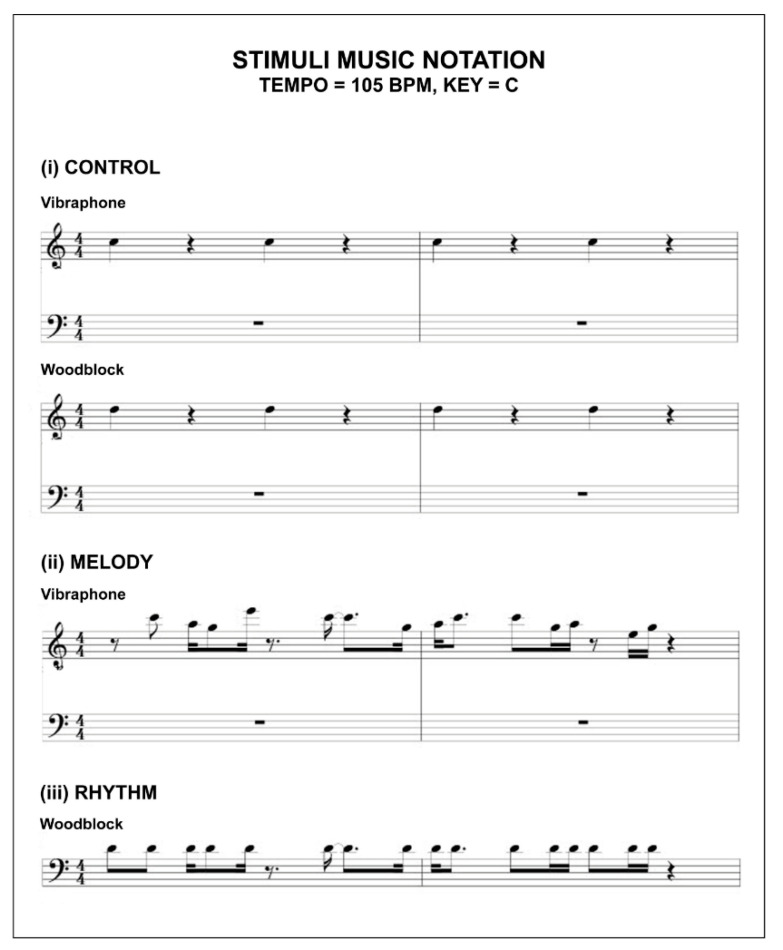
Musical notation for the control, melodic, and rhythmic stimuli.

The rhythmic stimuli retained the horizontal rhythmic contour and dynamic features of the melodic composition, however, it restricted the vertical tonal contour and timbre of the score to D6 and Woodblock, respectively. In so doing, the composition may be interpreted as the rhythmic counterpart to both the control and melodic scores through the increased rhythmic contour (comparative to the control) and salient percussive timbre (relative to the melodic stimuli). See Figure 5 for the stimulus musical notation.

### 4.5. Study Design

The study comprised of two Groups (A and B) that were required to undertake two test sessions (Session 1 and Session 2) conducted each week on a Tuesday and Wednesday morning, respectively, during the data gathering window. All participants were required to complete each test immediately after waking from an assigned stimulus that was supplied by the researchers as a replacement to their usual alarm sound. The waking stimulus was deployed on the participants preferred electronic device (desktop, laptop, tablet, or smartphone). Participants completed each test in their chosen location and at their typical time of waking. This method was selected to maximize the natural contextual environment in which subjects use auditory alarms for awakening in their daily routine, ensuring the ecological validity of the findings. Each test session required approximately 5–10 min to complete, being designed to collect high-value data whilst minimizing disruption to participants.

**Figure 6:**
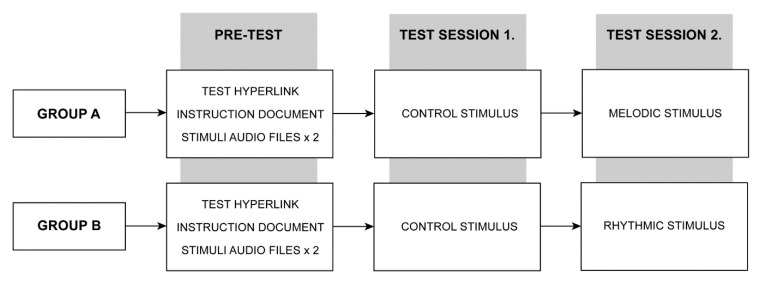
Experimental test protocol. See main text for full protocol design.

The participants (*N* = 20) were pseudo-randomly allocated and equally divided into two groups (Group A, *N* = 10; Group B, *N* = 10). Session 1 (Tuesday) required both groups to complete the study after awakening to the control stimulus. Session 2 (Wednesday) required Group A to complete the test following awakening to the melodic stimuli, and Group B the rhythmic stimuli. Figure 6 illustrates the study protocol.

At a minimum of twenty-four hours prior to commencing the study, each test group was supplied via email the test hyperlink, instruction document, and the test stimuli audio files. The hyperlink allowed each participant to access the test on the first day and resume the test the following morning. The design of the online study included timing nodes to safeguard against any participant attempting to access the study prior to (or between) each test date. The instruction document contained the pre-test preparation and the test procedure.

The pre-test preparation consisted of six steps for each participant to follow. These included the following: setting up the sounds on your device (Step 1), setting up the sounds as your two alarms (Step 2), setting the alarm volume (Step 3), testing the alarm sounds (Step 4), email link to the study (Step 5), and test preparation (Step 6). Steps 1–4 instructed each participant to first download the test stimuli onto their device, set the files as a separate alarm tone (each stimuli file is labelled corresponding to Tuesday or Wednesday), define the volume and disable the “rising volume” setting if applicable, test both stimuli for correct functionality, and familiarize themselves with each stimulus. Step 5 informed the participants that the hyperlink will direct them to the study and to check their email accounts as reminder emails will be issued prior to the second day’s test. Step 6 recommended that each participant has the relevant email open the night prior in preparation to activate the study link.

The test procedure information commenced by encouraging each participant to familiarize themselves with the protocol prior to undertaking the test and was followed by the process for each test session. The procedure for each session contained three steps for each participant to follow under the themes; upon waking (Step 1); beginning the test (Step 2); end of the session (Step 3).

The test battery for each study included an adapted brief psychomotor vigilance task (PVT, 3 min (Item 1)) [64], the Karolinska sleepiness scale (KSS, Item 2) [65,66,67], and two custom-designed Likert scales (sleep duration (Item 3); sleep quality (Item 4)). Session 1 also recorded demographic information (Gender (Item 5); age range (Item 6); hours typically slept (Item 7)). Please refer to the supporting material Table S1) for a transcript of the questionnaire for each test session. All responses were forced.

The PVT-B [68] is a validated 3 min variation of the popular 10 min PVT developed by Dinges and Powell [69], which records participant reaction time (RT) to random interval stimuli. Interpretation of this data is extrapolated into several performance metrics (i.e., mean RT, lapses, and false starts) as measures of behavioural alertness. Several research experiments have incorporated the PVT-B as an objective measure of a subject’s vigilance [70,71]. Benefits of using the PVT-B in this study’s context are that it can be undertaken remotely online and is intuitive for participants to perform [72]. The PVT-B is particularly suited to enquiries where the 10 min PVT is considered overtly time consuming [64]. Our adapted PVT-B was produced in the Gorilla task builder software [50] with respect to Basner and Dinges [68] and Basner et al. [64]. The test required each participant to either click a mouse controller, depress a keyboard key, or press a button icon on a screen (dependant on which device the participant nominated) immediately as a visual stimuli transitioned from one assigned colour to another. In our design, the subjects were instructed to respond as quickly as possible when a circular orange stimulus turned red. The interstimulus interval (ISI) between each coloured stimulus was randomized and varied between 1 and 4 s as specified by Basner et al. [64]. A timeout condition was included (≥1000 ms) to further ensure test duration was retained to a minimum while retaining responsiveness. During analysis, timeouts were interpreted as lapses with a 1000 ms duration. One of three statements were displayed following each response as a fixation substitute and to inform the participant of their continual performance. These were: (i) Too Quick! (false start); (ii) Great Work! (correct response); and (iii) Too Slow! (timeout). Each statement extended for 1000 ms and was deducted from the total (ISI) as previously described.

The KSS [65] is a subjective nine-point bipolar Likert scale measure of sleepiness that exists in two versions, A and B [73]. The original KSS A labelled the odd scales only (1 = extremely alert, 3 = alert, 5 = neither alert nor sleepy, 7 = sleepy but no effort to keep awake, and 9 = very sleepy and a great effort to keep awake, fighting sleep) while the KSS B [66] subsequently completed the even labels (2 = very alert, 4 = rather alert, 6 = some signs of sleepiness, and 8 = sleepy, some effort to keep awake). These two versions have been verified to be similar [73] and their results are comparable. In this study, we implemented version B. The instructions request the participant to indicate their “level of sleepiness during the 5 min before this rating by selecting the appropriate description”. We interpreted the response of this rating to reflect the perceived sleepiness of participants upon waking prior to the commencement of the PVT.

The custom-designed subjective measure of sleep duration (Item 3) is a self-report unipolar 14-point Likert scale. This design requests each participant to rank their sleep duration as “accurately as possible” from the fourteen options supplied. Each sleep time category is measured in increments of 0.5 h between either end categories of the scale (0–3 h and 9+ hours), for example, 0–3, 3–3.5, 3.5–4, 4–4.5, etc.

To record each participants’ subjective sleep quality, we developed a self-report five-point unipolar Likert scale. The scales options for selection contained, very poor, poor, average, good, and very good. The decision to design this single-item scale as opposed to utilizing the established Sleep Quality Scale (SQS, 5–10 min completion time) [74] or the Pittsburgh Sleep Quality Index (PSQI, 1 month reporting duration) [75], was to reduce potential time constraints of each participant. As the test was undertaken prior to each subject attending their employment obligations (if applicable), limiting the test duration was a factor in the design of this study. To gather demographic data of the participants’ typical hours slept each night we included a five-point unipolar Likert scale with the following options in hours, 0–3, 3–5, 5–7, 7–9, and 9+.

### 4.6. Statistical Analysis

With reference to Basner and Dinges [68], the PVT performance metrics recorded in this study were the mean RT, mean 1/RT (reciprocal response time or response speed calculated by dividing RT (ms) by 1000, then reciprocally transformed) [76], the number of lapses (≥500 ms), the number of false starts (RT ≤ 100 ms and responses prior to the red stimuli), and performance score (i.e., 1 minus the number of lapses and false starts divided by the number of valid stimuli including false starts) [68].

A planned sequence of five paired sample *t*-test’s [77] was completed to examine each vigilance metric between conditions for the respective groups (Group A: control vs. melody; Group B: control vs. rhythm). Cohen’s *d* was the effect size employed for the analysis of the paired *t*-test’s and was calculated by the mean of within-subject differences, divided by the standard deviation of the within-subject differences [31]. The Shapiro–Wilk test was applied to asses normal distribution of the data [78]. Datasets that rejected the normality hypothesis were analysed using the non-parametric Wilcoxon signed rank test [79].

The Wilcoxon signed rank test was also employed to analyse the median rank within-subject differences for the subjective measures (KSS, sleep duration, sleep quality) in each individual test group. The effect size selected for the analysis of each Wilcoxon signed rank test was calculated from the *z*-value reported by the test, divided by the square root of the number of observations recorded (i.e., *N* = 20) [80]. Additionally, percentage comparisons were undertaken for each subjective measure as an aggregate of both test sessions for each test group. An *α* = 0.05 was considered statistically significant for the analysis and power calculations. Raw data were initially tabulated for analyses in Microsoft Excel [81], and then imported to SPSS 26 [82] for statistical analysis.

## Figures and Tables

**Table 1:** PVT metrics of the paired sample *t*-test results for Test Group A (melody stimulus). The *p*-values less than 0.05 are represented by (*). The *p*-values less than 0.01 are represented by (**). See main text for full statistics.

Measure	Session 1. Control	Session 2. Melody	Test Statistics
Mean RT	*µ* = 504.21, *SD* = 107.02	*µ =* 475.84, *SD =* 84.43	*t(9)* = 1.44, *p* = 0.184, *d* = 0.455
Mean 1/RT	*µ* = 2.21, *SD* = 0.50	*µ* = 2.28, *SD* = 0.43	*t(9)* = −0.681, *p* = 0.513, *d* = −0.215
Lapses	*µ* = 24.20, *SD* = 18.29	*µ* = 19.40, *SD* = 17.10	*t(9)* = 2.94, *p* = 0.016, *d* = 0.930 *
False Starts	*µ* = 2.20, *SD* = 1.69	*µ* = 1.00, *SD* = 1.05	*t(9)* = 4.811, *p* = 0.001, *d* = 1.521 **
Performance Score	*µ* = 0.56, *SD* = 0.30	*µ* = 0.66, *SD* = 0.28	*t(9)* = −3.54, *p* = 0.006, *d* = −1.121 **

**Table 2:** Wilcoxon signed rank test results for subjective measures of Test Group A. See main text for full statistics.

Measure	Session 1. Control	Session 2. Melody	Test Statistics
KSS	*M* = 6.50	*M* = 6.50	*z* = 0.000, *p* = 1.000, *r* = 0.000
HOURS SLEPT	*M* = 10.50	*M* = 10.50	*z* = −1.186, *p* = 0.236, *r* = −0.265
SLEEP QUALITY	*M* = 3.00	*M* = 3.50	*z* = −0.378, *p* = 0.705, *r* = −0.085

**Table 3:** PVT metrics analysis results for Test Group B (rhythm stimulus). See main text for full statistics.

Measure	Session 1. Control	Session 2. Rhythm	Test Statistics
Mean RT	*µ* = 506.01, *SD* = 109.68	*µ* = 452.42, *SD* = 64.22	*t*(9) *=* 1.405, *p* = 0.193, *d* = 0.444
Mean 1/RT	*µ* = 2.23, *SD* = 0.40	*µ* = 2.38, *SD* = 0.35	*t*(9) = −1.058, *p* = 0.318, *d* = − 0.335
Lapse	*µ* = 20.40, *SD* = 14.92	*µ* = 17.40, *SD* = 13.45	*t*(9) = 0.530, *p* = 0.609, *d* = 0.168
False Starts	*M* = 1.00	*M* = 1.00	*z* = −1.30, *p* = 0.194, *r* = −0.291
Performance Score	*µ* = 0.64, *SD* = 0.25	*µ* = 0.68, *SD* = 0.23	*t*(9) = −0.421, *p* = 0.683, *d* = −0.133

**Table 4:** Wilcoxon signed rank test results for subjective measures of Test Group B. See main text for full statistics.

Measure	Session 1. Control	Session 2. Rhythm	Test Statistics
KSS	*M* = 7.00	*M* = 6.00	*z* = −1.699, *p* = 0.089, *r* = −0.380
HOURS SLEPT	*M* = 11.00	*M* = 11.00	*z* = −0.431, *p* = 0.666, *r* = −0.096
SLEEP QUALITY	*M* = 3.50	*M* = 3.50	*z* = −0.577, *p* = 0.564, *r* = −0.129

**Table 5:** Stimulus design overview.

Test Stimuli Design Overview		
Control	Melody	Rhythm
Metronomic contour	Metronomic contour	Metronomic contour
(Woodblock, Vibraphone)	(Woodblock, Vibraphone)	(Woodblock, Vibraphone)
	+	+
	Melodic contour	Rhythmic contour
	(Vibraphone)	(Woodblock)

## Supplementary Materials

Supplementary materials can be found at https://www.mdpi.com/2624-5175/2/2/17/s1. Table S1. Online questionnaire for each test session.
