# Supplementary material for: Auditory Countermeasures for Sleep Inertia: Exploring the Effect of Melody and Rhythm in an Ecological Context
Source: Clocks Sleep. 2020 May 29;2(2):208–24. doi: 10.3390/clockssleep2020017 (PMC7445849; doi:10.3390/clockssleep2020017)
Supplement: Supplementary file 1 [file clockssleep-02-00017-s001.pdf]

Auditory Countermeasures for Sleep Inertia: Exploring the Effect of Melody and Rhythm in an Ecological Context.

Supporting Information

Test Questionnaire

Stuart J. McFarlane[1*], Jair E. Garcia[1], Darrin S. Verhagen[2] , Adrian G. Dyer[1].

[1]School of Media and Communication, RMIT University, Melbourne, Vic, 3001, Australia.

[2]School of Design, RMIT University, Melbourne, Vic, 3001, Australia.

Test Session 1.

OPENING PAGE

Good Morning!

Welcome to the Waking Sound and Sleep Inertia Study!

Please follow the prompts carefully as they will guide you through the test.

By completing this test, you are giving your consent to participate in the study.

ITEM 1.

PVT Test

ITEM 2.

Please, indicate your sleepiness during the 5 minutes before this rating through ticking the appropriate description.								
1 = EXTREMELY ALERT	2 = VERY ALERT	3 = ALERT	4 = RATHER ALERT	5 = NEITHER ALERT NOR SLEEPY	6 = SOME SIGNS OF SLEEPINESS	7 = SLEEPY, BUT NO EFFORT TO KEEP AWAKE	8 = SLEEPY, SOME EFFORT TO KEEP AWAKE	9 = VERY SLEEPY, GREAT EFFORT TO KEEP AWAKE, FIGHTING SLEEP

ITEM 3.

Please indicate as accurately as possible how many hours you slept last night.													
**- 3**	3 – 3.5	3.5 - 4	4 – 4.5	**4.5 - 5**	**5 – 5.5**	5.5 - 6	6 – 6.5	**6.5 - 7**	7 – 7.5	7.5 - 8	**8 – 8.5**	8.5 - 9	9+

ITEM 4.

How would you describe your quality of sleep?				
VERY POOR	POOR	AVERAGE	GOOD	VERY GOOD

ITEM 5.

What is your age range?				
18 - 29	30 - 39	40 - 49	50 - 59	60+

ITEM 6.

What is your gender?			
MALE	FEMALE	X (INDETERMINATE/INTERSEX/UNSPECIFIED)	PREFER NOT TO DISCLOSE

ITEM 7.

How many hours do you typically sleep each night?				
0 – 3	3 - 5	5 - 7	7 - 9	9+

CLOSING PAGE

You are all done!

If you are feeling drowsy and must operate any heavy machinery, drive or complete tasks of a consequential nature, the research team recommends that you wait until your drowsiness subsides.

See you tomorrow!

Test Session 2.

OPENING PAGE

Good Morning!

Welcome back!

Please follow the prompts carefully as they will guide you through the test.
Please begin by pressing the resume button.

ITEM 1.

PVT Test

ITEM 2.

Please, indicate your sleepiness during the 5 minutes before this rating through ticking the appropriate description.								
1 = EXTREMELY ALERT	2 = VERY ALERT	3 = ALERT	4 = RATHER ALERT	5 = NEITHER ALERT NOR SLEEPY	6 = SOME SIGNS OF SLEEPINESS	7 = SLEEPY, BUT NO EFFORT TO KEEP AWAKE	8 = SLEEPY, SOME EFFORT TO KEEP AWAKE	9 = VERY SLEEPY, GREAT EFFORT TO KEEP AWAKE, FIGHTING SLEEP

ITEM 3.

Please indicate as accurately as possible how many hours you slept last night.													
**- 3**	3 – 3.5	3.5 - 4	4 – 4.5	**4.5 - 5**	**5 – 5.5**	5.5 - 6	6 – 6.5	**6.5 - 7**	7 – 7.5	7.5 - 8	**8 – 8.5**	8.5 - 9	9+

ITEM 4.

How would you describe your quality of sleep?				
VERY POOR	POOR	AVERAGE	GOOD	VERY GOOD

CLOSING PAGE

You are all done!

If are feeling drowsy and must operate any heavy machinery, drive or complete tasks of a consequential nature, the research team recommends that you wait until your drowsiness subsides.
Please press the finish button to submit your results.

On behalf of the research team we would like to thank you for taking time to take part in this study!
